# The Caregiving Experiences of Fathers and Mothers of Children With Rare Diseases in Italy: Challenges and Social Support Perceptions

**DOI:** 10.3389/fpsyg.2019.01780

**Published:** 2019-08-05

**Authors:** Paola Cardinali, Laura Migliorini, Nadia Rania

**Affiliations:** Department of Educational Science, University of Genoa, Genoa, Italy

**Keywords:** caregiving, social support, rare disease, gender differences, parents’ perception

## Abstract

Family caregiving is a growing phenomenon with the increased prevalence of chronic illness and shorter hospitalizations. Rare diseases pose significant challenges not only to patients living with these kinds of pathologies but also to those who care for these patients. The caregiving role has specific characteristics. The present work aims to increase knowledge of the challenges that are common or specific to fathers and mothers of children diagnosed with a rare disease. Moreover, the paper analyses the kinds of social support they experience according to gender. A descriptive study was conducted using grounded theory methodology. A semi-structured interview with open-ended questions was conducted with 15 parents of children with a rare disease. The interview was organized into three main areas: personal experiences in caring for a child with a rare disease, family changes and perceived social support. The transcriptions were analyzed using NVivo 11 software. From data analysis, themes emerged regarding the challenges shared by fathers and mothers, but some aspects also emerged that were gender-specific. The analyses of differences between mothers’ and fathers’ narratives showed that there is a specific experience of the impact that caregiving has on parents’ relationships with their jobs and on their worries. Self-help group is the main source of social support for all respondents. We discuss these findings in relation to possible appropriate specific interventions and support for family caregiving.

## Introduction

Caring for a child with a disease is a family endeavor; therefore, family caregiving is a growing phenomenon in countries throughout the world, as the prevalence of chronic illness and the frequency of shorter hospitalizations increase (Revenson et al., 2015). The illness of a family member may drive other family members into a new life situation, in which the need to provide care redefines one’s relationship with others, daily routines and perceptions of the future. As caregivers move into their role, they may experience a change in usual practices and a focus on the challenge of being a caregiver. Family caregivers may also perceive role ambiguity (Gibbons et al., 2014), which may even more occur when they cope with challenges related to a rare disease.

Rare diseases are a large heterogeneous group of illnesses that require long-term care. Rare diseases are important public and social issues that pose significant challenges to communities (Schieppati et al., 2008). They encompass severe diseases with very unfavorable prognoses. The definition of a rare disease is arbitrary and varies according to the geographical area; it depends on the epidemiological characteristics and on the number of patients in a territory. Europe defines rare diseases as those that affect less than 1 in 2,000 persons. The number of known and diagnosed rare diseases varies between 7,000 and 8,000 in Italy (Taruscio, 2009). A national institutional registry of rare disease patients was established in Italy in 2001 and is administered by the National Center for Rare Diseases of the National Institute of Health (Taruscio et al., 2014). According to the Orphanet network, 2 million patients in Italy suffer from rare diseases, 70% of whom are pediatric patients.

The Fifth Report on the condition of the Rare Patient in Italy (2019) highlights some strengths that characterize the country: 17 Regions have defined a Regional Rare Diseases Plan, moreover Italy is at the first place in terms of number of health care providers members of the European Reference Networks. Compared to previous years, there has been an increasing in the quality and the coverage of surveillance systems and a marked increasing in neonatal screening for hereditary metabolic diseases. Another positive element is the active participation of people with rare diseases and their delegates in decision-making groups.

However, there is a territorial irregularity in access to health and social services and a lower presence of Italian research groups in rare disease projects included in the Orphanet platform. This consideration supports the importance of implementing research projects in this area in the Italian context.

Rare diseases pose particular challenges not only to patients who are affected (Stoller, 2018) but also to those who care for these patients. The literature has examined in particular the challenges associated with living with a rare disorder as a child or as an adult, while it has neglected the role of caregivers, who nonetheless undergo profound changes related to caring for a child with an illness.

A recent review (von der Lippe et al., 2017) highlights three main research areas focused on patients with a rare disorder: first, the consequences of living with a rare disease, which includes constraints, limitations, and a psychological impact, specifically related to uncertainty and coping strategies; second, the social aspect of living with a rare disease, which includes secrets about diagnosis, stigma and isolation, a desire for normalcy, and the need for support; and third, experiences with the health care system, which comprises a lack of knowledge, the turnover of health professionals, and expertise in their own diagnosis. However, the literature does not focus on the psychological and social impact that can characterize even those who take care of a patient with a rare disease. Despite this interest in patients with rare diseases, there has been little investigation into the overall experiences of parents and caregivers of children with complex rare disorders (Currie and Szabo, 2018). Nonetheless, these diseases have a considerable impact on the quality of life of not only patients but also their main caregivers, who can perceive a very deleterious impact on their social, professional and family life (Michalík, 2014; Tejada-Ortigosa et al., 2019). As is the case with most childhood chronic conditions, rare diseases impact the whole family, especially with respect to normal family routines that serve an important scaffolding function for the well-being of children (Emiliani et al., 2010; Hammons and Fiese, 2010; Migliorini et al., 2011, 2016; Potì et al., 2018) as well as with respect to increases in the family’s financial burden (Dogba et al., 2013). For these reasons, the need to adopt a family centered approach to children’s chronic conditions has been repeatedly emphasized (American Academy of Pediatrics [AAP], 2003; Migliorini and Rania, 2016). This article therefore explores a topic that has been little examined in the literature about rare disease.

Parents of children with chronic health problems must adapt to new roles, reorganize their lives and cope with increased care demands, but parents of children with rare diseases must face further complications: the diagnosis, which may be delayed or not specifically defined; a lack of support groups, which, even if present, are based in geographically dispersed areas; and a scarcity of medical skills and health resources (Jaffe et al., 2010; Pelentsov et al., 2014). Parents can experience limited collaboration and integration of support and resources in the overall medical plan for their child (Currie and Szabo, 2018). Some studies underline that parents are burdened with the additional role of the care coordinator in the health system to improve care continuity (Budych et al., 2012; Baumbusch et al., 2018). Anderson et al. (2013) highlighted that parents felt that their experiences could be improved with better coordination of care and the introduction of electronic health records accessible by the many different health professionals with whom they interacted. These impacts on families have not yet been systematically explored in Italy. A lack of studies exists that explore the supportive needs of parents of a child with a rare disease, and in Italy, only a recent study (Zagaria et al., 2018) has explored parents’ changing roles according to child disease. This study shows that mothers of children with rare disease refer an high emotional commitment, whereas fathers depict the disease as an obstacle to a “normal” life.

In the literature, the most investigated tasks refer to the need for general medical information regarding the illness of one’s child, financial worries and healthcare costs, parents’ feelings of loneliness and isolation, and the physical and emotional burden of caring for a child with a rare disease (Glenn, 2015; Pelentsov et al., 2016). Previous studies have described a state of “pilgrimage” that takes place among various health institutions and specialists due to the lack of structured health policies and centers for rare diseases (Lopes et al., 2018). Other studies underline that parents must assume the role of ‘expert’ (Brewer et al., 2008; Pelentsov et al., 2015), since the rarity of the disease can increase the likelihood that professionals are not specifically competent in the subject matter. The diagnosis represents a turning point that could enable families to start adjusting to their new normality (Germeni et al., 2018), but this moment often requires a long period of time. Furthermore, being parents of a child with a rare disease has been shown to have profound implications on the physical and psychological health of the parents, particularly among mothers, and to negatively influence one’s relationship with his/her partner (Pelentsov et al., 2015).

Social support represents a key variable associated with optimal adjustment to a chronic illness (Helgeson and Zajdel, 2017; Vargas Bustamante et al., 2018). According to the classical contributions of Cohen and Wills (1985), the literature has demonstrated that social support could buffer the relationship between stress and well-being. The stress and coping perspective suggests that social support buffers the negative effects of stress; received support is thought to help people cope, perceive support, and alter perceptions of potentially threatening situations. Isolated people could have greater difficulty in addressing stressful situations and could be more likely to develop diseases related to stress, psychosomatic pathologies, problems with the circulatory system and sometimes predispose them to dangerous behaviors that negatively affect their health (De Piccoli, 2014). Therefore, a network of social support plays a crucial role in maintaining control over chronic conditions (Chen et al., 2018). Social support may be particularly important for high-risk groups of parents (Ergh et al., 2002), such as those who must care for a child with a rare disease (Bogart et al., 2017).

Social support has been referred to as a multidimensional construct comprising different aspects. Vaux (1988) states that it can be actual (behaviors performed by the support network) or perceived (assessments of the availability and quality of support by the individual). The first dimension of social support concerns actual support received, such as the type and amount of services or the type and amount of supportive interactions, and can be divided into emotional support (e.g., displays of intimacy or encouragement), informational support (e.g., advice, guidance, and suggestions), esteem support (e.g., that designed to strengthen an individual’s sense of competence), and tangible support (e.g., concrete assistance, such as financial help) (Vaux and Harrison, 1985). The second dimension concerns the perception that support is available; nevertheless, this support is actually required or received (Barrera, 1986).

The support system can be informal or formal. The informal system includes relationships with people with whom one has a higher degree of familiarity and with whom one shares basic principles, ideas, interests, and social objectives. Within this system, we can distinguish between two subcategories represented by the primary network (family members and closest friends) and the secondary network (groups and associations born spontaneously to satisfy the needs of the members or the social context). Both provide, to different degrees, emotional closeness, affiliation and protection. The formal system is represented by the set of professionals who work in the contexts of care, rehabilitation or psychosocial intervention and who take care of the specific needs of each community (Zani, 2012).

Often, parents of children with rare disorders feel lonely, ill-supported by institutions, and often are interested in being part of support groups or in actively participating in organizations for patients suffering from rare diseases, thus allowing them to obtain increased support, understanding and dissemination of information compared to what is provided by medical personnel, who are often not sufficiently informed (Aymé et al., 2008). Sometimes parents complain about professionals’ lack of empathy (Čagalj et al., 2018), and they have a consequent low perception of formal support.

In this framework, it could be clearer why it is so important for parents to interact with and receive informal social support from other parents who care for a child with a rare syndrome (Kerr et al., 2004; Coffey, 2006; Duffy, 2011). According to the literature (Čagalj et al., 2018), the need for information about a child’s condition and the need for health professionals’ support are the most common necessities of parents caring for a child with a rare disease. Informational and emotional support can be found from parents also connecting with peers online (Glenn, 2015). When social support is derived from a self-help group, it can address both the psychological function of emotional support and practical assistance and the social function, which is oriented toward raising awareness and changing the community within which the group is inserted. Social psychology offers a vast and solid experimental literature that has highlighted, as in difficult conditions, the absence of a reference point, relationships with others, a lack of judgment and the provision of availability, all of which could help people develop highly functional adaptive strategies (Dennis, 2003; Albanesi, 2004). Therefore, informal peer support from other parents emerged as a key resource for this population (Pelchat et al., 2007; Baumbusch et al., 2018); it improves their emotional regulation in relation to the continuous challenges posed by living with children suffering from chronic diseases, serving as a protective factor and reducing the accumulated stress (Benn and McColl, 2004).

Formal support in the school context seems to be determined by a close mother-teacher relationship (Čagalj et al., 2018); this finding seems reaffirm the central role of women in caregiving. This gender inequities could be perpetuated by an assumption that family caregiving is naturally linked to women’s role and identities (Migliorini and De Piccoli, 2019). The experience of male caregiving has been largely neglected in the literature (Denby et al., 2014). The specific needs of fathers have not been explored in depth in previous studies focusing on parents of chronically ill children (dos Santos et al., 2017). Studies that have examined gender differences in caregiving have shown greater care and support provided by women (Sharma et al., 2016; Palacios-Ceña et al., 2018), along with greater levels of stress, depressive symptoms and poorer well-being and overall health (Del-pino-casado et al., 2012; Li et al., 2013). This finding appears to be in line with traditional societal norms in which men are seen as responsible for financial stability while women are assigned the role of caregivers. In a recent study (dos Santos et al., 2017), mothers complained about being overloaded and the difficulty of reconciling different functions, while fathers complained about not being able to be present with the patient as much as they would like; they also express worries concerning the other siblings. The culturally shared gender role construction entrusts women with the duty to take responsibility for care at the cost of sacrificing some aspects of their lives (De Piccoli, 2015). Male caregivers conceive of a demonstration of their own vulnerability as inappropriate for men (Rollero, 2019). Bullock (2007) suggests that because of traditional values, men who assume the role of caregiving may be less inclined to ask for help. The understanding of gender difference in caregiving is untapped in literature about rare disease. However, it could be useful a more in-depth analysis of gender differences that could enhance the clarity of parents’ experiences in caring for children with a spectrum of rare diseases, especially with respect to their perceptions of social support.

The present work aims to increase knowledge on the impact that rare disease could have on caregiver’s perception of daily life, feelings, behaviors and social support.

In particular we hypothesize to underline:

- The types of challenges that are common in narratives of fathers and mothers of children diagnosed with a rare disease;
- The types of challenges that are gender-specific for father and mothers;
- The types of social support caregivers experience, with particular attention to possible differences between fathers’ and mothers’ perceptions.

## Materials and Methods

### Participants

A convenience sample is made up of people who participated in a self-help group for parents of children with rare diseases. The participants were 15 Italian parents (7 fathers and 8 mothers) of children diagnosed with a rare disease. A brief description of the main symptoms that are typical of childrens syndromes are presented in Table 1.

**TABLE 1:** Rare syndromes main symptoms.

**Rare disease**	**Main symptoms**	**Prevalence**
Aicardi syndrome	Agenesis of the corpus callosum, characteristic chorioretinal lacunae, infantile spasms, characteristic facies, microcephaly, periventricular heterotopia, microgyria, ventricular dilatation, porencephalic cysts, axial hypotonia and appendicular hypotonic hypotonia.	Unknown
Angelman syndrome	Severe mental retardation, characteristic facial dysmorphism, absence of speech, rice crises associated with stereotyped hand movements, microcephaly, macrostomy, maxillary hypoplasia, prognathism and neurological disorders, ataxia, seizures, hyperactivity, sleep disorders	1–9/100 000
Arginine succinic aciduria	Hyperammonemia associated with vomiting, hypothermia, lethargy and feeding difficulties, behavioral abnormalities and/or learning difficulties, liver dysfunction.	1–9/100 000
Chromosome 22 Ring	2–3 toe syndactyly, autistic behavior, azoospermia, bulbous nose, delayed speech and language development, developmental regression, dolichocephaly, generalized hypotonia, global developmental delay, impaired pain sensation, inappropriate behavior.	<1/1 000 000
Fryns syndrome	Facial dimorphisms, congenital diaphragmatic hernia, pulmonary hypoplasia and distal limbs, associated with other malformations in various combinations.	Unknown
Goldenhar syndrome	Craniofacial microsomia, dermoid ocular cysts, spinal anomalies, epibulbar dermoid tumors, preauricular appendages and ear malformations.	1–9/100 000
Klinefelter syndrome, 49 XXXXY	Hypogonadism, reduced fertility, tendency to obesity, reduced language development, with communicative problems.	1–9/100 000
Lesch-Nyhan syndrome	Dysarthria, dysphagia, opisthotonus, spasticity, hyperreflexia and plantar reflexes in extension. Patients usually have mild to moderate mental retardation. At the time of the dental eruption obsessive-compulsive self-harm (biting of lips or fingers) may occur. Aggressive behavior can be maintained toward family or friends (spitting, offensive language).	1–9/1 000 000
Mucolipidosis, type III	Pseudo-HurlerIan polydistrofia, facial dysmorphism, corneal opacity, learning difficulties.	Unknown
Prader-Willi syndrome	Hypothalamic-pituitary anomalies associated with severe hypotonia in the neonatal period and in the first 2 years of life; hyperphagia, which results in the risk of morbid obesity in infancy and adulthood, learning difficulties and behavioral problems or serious psychiatric problems.	1–9/100 000
Rett syndrome	Apparently normal development in the first 6–18 months of life and, subsequently, loss of gross and fine motility already acquired, loss of the ability to interact and socialize, severe cognitive deficit, stereotyped movements of the hands.	1–9/100 000
Wolf-Hirschhorn syndrome	Characteristic facial cranial signs, delay of prenatal and postnatal growth, cognitive impairment, severe delay of psychomotor development, convulsions and hypotonia.	1–9/100 000

*https://www.orpha.net.*

Even if the different syndromes present a variability in symptoms, participants share the condition of rarity that characterizes their children’s disease.

All participants were married. The mean age of respondents was 52.12 years for mothers and 57 years for fathers. The 33% hold a degree, the 40% have a diploma and the 27% have a secondary school certificate. A total of 50% of women were not working, while 71% of men were employed. In three circumstances, the child was suspected by health professionals of having a rare disease that had not been formally diagnosed. The majority of children (87%) had siblings. With respect to the children’s birth order, the first child of five families had a rare disease, the second child of seven families had a rare disease, the fourth child of one family had a rare disease, and both the first and second children of two families had the same rare disease.

### Materials

In addition to the collection of demographic data (age, gender, educational level, and current employment status), a semi-structured interview with open-ended questions was conducted among parents of children with a rare disease. The interview was organized in three main areas: the personal caregiving experience for a child with a rare disease, family change and perceived social support. The interview opened with a joining phase, in which the participant became familiar with the interviewer, trying to adapt to each other, feel at ease, and overcome any embarrassment that could alter the meaning of the communication. In this first part, socio-demographic data and some information on syndromes were collected.

For each area described above, a few key questions guided the interview: Can you tell me your story since (the child’s name) was born? How did you experience the diagnosis? How did you take care of (the child’s name)? (personal caregiving experience); How did the family change when (the child’s name) was born? (family change); Who helped and supported you? How did the group help you? (perceived social support).

Although there was a fixed and common track for everyone, the conduct of the interview may vary based on the answers given by the interviewee and based on the individual situation. The interviewer developed some topics that emerged spontaneously during the interview, if useful for understanding the participant’s experience. The interview lasted approximately 60 min.

### Procedure

The project was presented to parents who participated in a self-help group for parents of children with rare diseases. Researchers were introduced to the caregivers via a coordinator of the self-help group that meets in northwestern Italy. A subjective report of their caregiving experiences was collected from each participant (mother or father). Participants were asked to complete a brief socio-anagraphic schedule and to provide informed consent. Interviews were audiotaped and transcribed verbatim. All participants took part on a voluntary basis and signed a consent form describing the study. This form assured them that the information they provided was for research purposes only and was confidential and that they could discontinue the interview at any point.

### Data Analysis

A grounded theory approach (Glaser and Strauss, 1967) was selected for the present study. We used the objectivist approach because of the descriptive and explorative nature of the aims. The transcripts were analyzed with an iterative process of the collection and examination of data (Charmaz, 2005). Data were compared from common teams using NVivo11 software. The interview transcripts were coded privately and independently by two researchers using a codebook and coding scheme for emerging themes or recurring domains of meanings across the narratives (Lofland and Lofland, 1995; Rossman and Rallis, 1998). All disagreements were discussed, and a code was agreed upon. The software was used to organize the coded statements into nodes containing similar concepts and hierarchies of categories and subcategories. The data analysis generated graphical representations of the main topics. These models allow to explore the connections between nodes visually and will be presented in figures. The quotes inserted in the results were chosen from narratives to best represent the core emerging themes. The quotations were checked carefully to ensure that the meanings were preserved in the form in which they were presented by the participants.

## Results

### The Shared Challenges Among Fathers and Mothers

**FIGURE 1:**
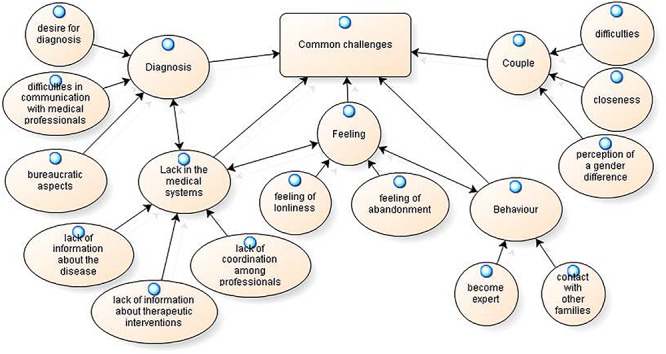
Common challenges experienced from fathers and mothers.

**FIGURE 2:**
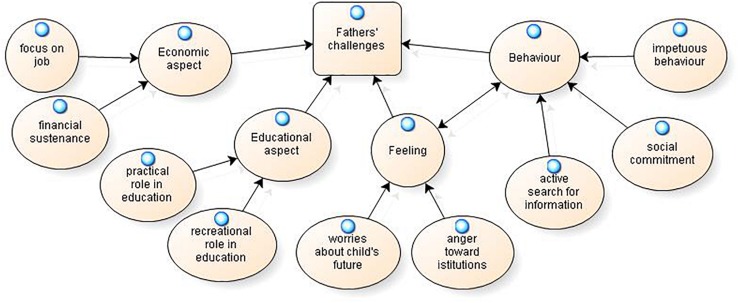
Fathers’ challenges.

**FIGURE 3:**
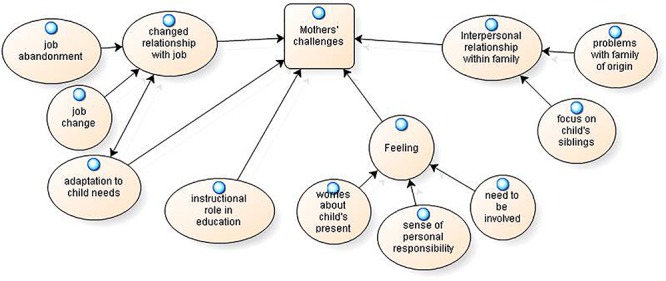
Mothers’ challenges.

The experience of being a caregiver of a child with a rare disease impacts parents in different ways. The analysis of the narratives has revealed some categories that appear to be common in mothers’ and fathers’ narratives and that will be illustrated in Figure 1. Some themes were present only in the interviews of the fathers and will be presented in Figure 2. Other categories are present only in the mothers’ interviews and will be presented in Figure 3.

The common challenges that emerge from both mothers’ and fathers’ narratives could be organized into five macro-areas: the diagnosis, the lack of a medical system, the feeling, the behaviors and the couple (Figure 1).

The first area pertains to the diagnosis, which includes the desire for it, the difficulties in communication with professionals about it and the bureaucratic aspects related to not having a diagnosis.

The desire for a diagnosis represents a central theme both for fathers and mothers; both emphasize the long wait for it. Some respondents stated the following:

“*No one had answers*; *also*, *we have been waiting for genetic research for more than a year*” (mother); “*We went on two and a half years*, *and*, *at the end*, *they gave us the diagnosis*” (father); “*I wanted to have an answer*, *and I desired a diagnosis*, *first of all*, *for S.* (*daughter*), *but now she is 23 years old*” (father); “*The diagnosis*, *for me*, *was like having a manual of instructions*, *finally*, *that I’ve been waiting for a long time*” (mother).

However, even when the diagnosis was determined, parents reported many unknowns about the disease and how to proceed with treatment, as reported above. The issue of diagnosis is frequently associated with a difficulty in communicating with medical professionals: “*Even after I told him that I work and that he could talk to me normally*, *nothing came to me*; *I received medical information and explanations*, *but when they finished speaking*, *it was as if they hadn’t said anything*” (father). “*The fact that someone put himself at our level and said this thing*, *he came to talk to us*, *and then he said*, *‘In my opinion*, *it is so*, *but he has atypical characteristics’…we needed someone to talk to us in this way*” (mother).

Some difficulties concern the bureaucratic aspects related to the absence of a clear identification for the pathology, as described by this mother: “*I did not have a code*, *because she had a severe psychomotor delay with suspected Rett syndrome*, *and there was no code to put on the form*” (mother). With respect to requests to institutions, the following opinion was shared: “*Every year*, *a fight for things occurs that should be automatic*; *you shouldn’t go around knocking on doors*, *writing letters to everyone for hours of school support*” (father).

The second area concerns the lack in the medical systems, including the lack of information and the lack of coordination among professionals.

The perceived lack of information is related to both of the following:

• Lack of information about the disease: “*It seemed that* (*the doctor*) *didn’t know how to do it*, *and maybe it’s embarrassing*” (father); “*After the diagnosis*, *we were still wandering for a year with a paralyzed child and a thousand questions: What happens? Will he live? Will he be twenty? And no one gave us information*” (father).
• Lack of information about therapeutic interventions: “*There was no indication of what could have been*, *not a solution*, *but some proposals*, *I don’t know*, *of rehabilitation*, *we hoped*, *because one hopes…one needs not to have answers*, *I repeat*, *but rather some indications*, *some advice on therapy*” (mother).

This theme seems to be related to the lack of coordination among professionals described by both fathers and mothers, which increases their caregiving burden: “*There is no doctor who coordinates specialist checkups or who can guide us in finding the right person to solve the specific problem*” (mother); “*I’m going crazy*, *then*, *to look for all the specialists to do these certifications*” (father); “*They are subjected to continuous visits*, *without purpose*, *and they visit them 10*, *20*, *30 times—stop! It is also humiliating* [*…*] *to lack projects of socio-sanitary collaboration*” (father); “*The doctors do not talk to each other*” (father).

This lack of knowledge and the difficulties in dealing with the healthcare system could generate emotional reactions in parents, including feelings of abandonment and loneliness.

Parents describe the feeling of loneliness*:* “*This made us feel lonely and with so much fear*” (father); “*Our problem is that of isolation*, *that is*, *the rare patient is isolated*, *in a context of homologated disabled people. We are not homologated*; *we are different*, *lonely*” (father); “*His suffering could depend on wrong choices and different opinions*; *this is what made me feel lost*, *more alone*, *both me and F.* (*husband*)” (mother).

Another common emotion is the feeling of abandonment as a significant problem in their life: “*A very strong problem that we faced was feeling abandoned on this path*, *after the birth indications that gave us information on where and by whom T.* (*son*)*’s problems would be corrected*; *we realized that they were not the right ones*” (father).

Another area that emerges from narratives is the behaviors that parents put into practice, such as the learning of specific knowledge about the disease that transforms them into experts or contact with other families.

Both fathers and mothers told us that over time, they had become experts on the disease and treasured this experience: “*I saw a particular book and immediately bought it*; *I heard music and bought the cassette*; *I created opportunities*, *new ideas for physical therapy*” (mother); “*I am the president of the association X*, *which aims*, *above all*, *to inform doctors about the pathology and the guidelines*” (father).

The possibility of having contact with other families appears to be extremely beneficial, as it provided emotional and practical support, reducing feelings of social isolation: “*We hugged each other and we started crying together*, *and this embracing and crying together was of mutual comfort*; *I knew that I had nothing to say because I was living a situation in which I realized that words served no purpose*, *and she had nothing to say*, *only great pain*” (mother); “*I said*, *‘Get together in some groups because if you are alone you do not combine anything’* “ (father); “*If you belong to a group with other families*, *you know that you are not alone*, *that together with you there is always a nice group of people…when you protest…let’s all go together*” (father).

Finally, parents highlighted a macro-area that concerned the couple, including problems and resources and the differences perceived between the partners about caregiving.

A common issue is couple difficulties: “*As a couple*, (*the disease*) *made us experience different storms*” (mother); “*Even in-home relationships*, *a bit of tension surely came out over time*; *there was some discussion in general too*, *which could have been avoided…*” (father). However, this experience seems to have also created closeness within the couple: “*We also know how to love each other and welcome each other into our powerlessness*” (mother); “*My wife and I have overcome conflict*” (father).

Both men and women reported the perception of a gender difference in the impact of a child’s rare illness on one’s life; particularly, the idea that the father maintains normality in his life is the most shared aspect: “*Each of us reacted differently…I said to my husband*, *‘Your life has not changed*, *there are your friends*, *your work*, *the outburst of the ball game’*; *nevertheless*, *I identified with this role…I lost all my friends*, *and I had left all things*” (mother); “*It was a tragedy*, *more for B* (*wife*) *than for me*, *because a mother is certainly more connected*, *more attached to the child…to take her away from the hospital*, *I had to fight*, *and she cried* [*….*] *I fell into a state of unconsciousness. I didn’t realize that I was living such a normal life*” (father).

Perceptions related to cultural differences with respect to family roles were also highlighted by one mother: “*To see a father who did the work of a mother moved me*.”

### Fathers’ Challenges

A main objective of our work was to identify what kinds of challenges were gender-specific.

Figure 2 shows a graphical representation that summarizes the challenges present only in fathers’ experiences. We describe these issues by quoting sentences from the men’s transcriptions.

Fathers’ challenges could be organized into economic aspects, educational aspects, feelings and behaviors, as described below.

Economic aspects include the importance attributed to one’s job and to the financial subsistence of the family. A first theme that emerges is the focus on the job, which requires a substantial amount of time in fathers’ lives, as well as a concrete commitment: “*I am leaving for Bologna to organize a course* [*…*] *for 1 week per month*, *I live in Bologna*, *and for this reason*, *my family suffers*;” “… *then I always look for ways to work*, *to work more than you can* [*…*] *I try to build family tranquility even at the economic level*.” This issue could be associated with the idea of having to address the financial sustenance of the family: “*I wanted supports so that I could go to work*, *because I was the only economic support at home*; *my wife is a housewife*.”

Concerning the macro-area related to educational aspects, fathers report a practical role: “*When I have some time*, *I always try to bring him here and there*; *he always wants to go out*, *he wants to do something…*;” “*Maybe I take him to the pool*, *he goes to the pool*, *I try to be present.*” Alternatively, fathers play a recreational role: “*As soon as I can*, *as soon as he is well*, *he wants to ride a bicycle*, *but with a bicycle on wheels*; *I take him on a bicycle*;” “ *She was very happy if I took her out to the restaurant…mixed fry*, *ravioli*, *she was really happy. I thought it would hurt her*, *but it was one of the few pleasures in her life.*”

The third macro-area concerns the feelings that fathers specifically report, including worries and anger. In particular, fathers seem worried about the future of their children: “*I know that when T.* (*child*) *grows up*, *he will have to address bigger problems than he does now*, *such as his inclusion in society*, *in the world of work…*;” “*When you become an adult…you disappear*; *this is already happening to him because he is slowly being taken away from some environments.*”

The relationship with organizations is characterized by emotions of anger toward institutions: “*My approach with institutions has always been one of anger*, *because the most obvious things are sometimes denied*;” “*I was a bit pissed off* (*excuse the sentence*, *I have to say this*); *I always got angry with everyone*, *the whole world was my enemy*;” “*Maybe it was me who was also angry with the institutions*, *and this anger came back to me with more anger.*”

Finally, fathers describe some behaviors that they put into practice in relation to caregiving for a child with a rare disease. These behaviors are the active search for information, the commitment to social activities and the general predisposition toward impetuous behavior.

A frequent behavior that fathers report is the active search for information about professionals, associations and other families who experience the rare disease: “*We have been looking for those who*, *for the illness of my son*, *are the most competent in the world. Dr. F. of Paris came to our conferences and explained everything clearly*;” “*I went to look for* (*the information*) *through the experience lived by others*, *so I could know where and from whom I could access this type of intervention*;” “*I found this association for Klinefelter syndrome*, *which I approached just to see if there were other cases like him*;” “*I contacted the president of the association* [*…*], *and he explained to me what I had never heard from doctors*;” “*I looked for other parents to understand the pathology well and then make choices and evaluate*, *because to decide*, *we must also evaluate*.”

In fathers’ narratives, a social commitment through active participation in the social context emerges: “*We have organized conferences with experienced doctors*; *most are not Italian* [*…*] *we have exported*, *given to others…in the sense that we have told our experience and collected others*, *put them together*, *collected them in a databank…*,” “*I participate in the regional table of rare diseases*; *I am a point of reference for my region*;” “*We succeeded in a battle to have the center settled*;” “*With the group*, *we met everyone*; *we met the politicians.*”

Finally, fathers frequent report impetuous behavior related to their feelings of anger: “*I immediately arrived at the fight*, *I left and I couldn’t take it anymore* [*…*] *I took A.* (*son*), *who had stopped*, *and we left*;” “*I went to a hospital complaints office*, *I made complaints*, *I called the doctor*, *I sparked half a mess.*”

### Mothers’ Challenges

The challenges associated with caregiving for a child with a rare disease that were present only in women’s experiences are shown in Figure 3. Additionally, for mothers, it is possible to describe other areas that emerge: relationship with one’s job, adaptation to the child’s needs, role in education, feelings and the family system.

A first aspect that women highlight is a changed relationship with one’s job, which is considered an activity that absorbs substantial time and is not satisfying. This area includes the possibility of abandoning one’s job or changing it.

Many mothers speak about job abandonment: “*Everything was upset*; *I left the job*, *so we had great difficulties…*;” “*I stopped teaching a dozen years ago to take better care of M.* (*son*), *who*, *with his serious pathology*, *absorbed a lot of time but also energy*.” Other mothers changed their jobs: “*I continued to be an engineer*, *which I did not like*, *as life can massacre you…so I stopped massacring myself*, *and now I am a teacher* [*…*] *I had to change jobs because I had to develop my creativity to raise a child like D.*”

This change appears to be associated with growing adaptation to children’s needs; it involves daily routines and rhythms of life: “*So we began to live with him*, *with his times*, *his rhythms*;” “*I always accompanied her*, *I changed shifts and went…*”

Mothers perceive that they have taken an instructional role in their relationship with their sons: “*My husband was the playful part*, *he played with him*; *I*, *as I said*, *fell into this role of teacher*, *which still has not abandoned me*;” “*She knew the tables very well because I taught them to her*;” “*I prepared her at home*, *in fact*; *then they were all amazed and said ‘Damn*, *what exams she did!’*”

Another macro-area concerns mothers’ feelings, including worries about the child’s present, a sense of responsibility for the child’s disease and the need to be involved among professionals.

The worries about the child’s present is an important theme for mothers: “*The most important thing is not what he can do*, *what he is able to do*, *or how he can go on*, *but that he is really happy…*;” “*I am satisfied when I see him* (*son*) *peaceful*;” “*We eventually realize that what he would like is a friend*, *and you can’t buy a friend*, *you can’t pay for it*, *and this is what D.* (*son*) *suffers*.”

In many interviews, the perception of women of having a personal responsibility with respect to the pathology of their child emerges: “*I thought myself responsible anyway because I had not been able to give birth well*;” “*Maybe it wasn’t the time to have another child*, *maybe if I did it before or after…*;” “*I felt blamed*, *but maybe it’s more my feeling.*”

In women’s verbalizations, the need to be involved in their children’s schools or rehabilitative projects is perceived as a challenge that modifies personal identity and behaviors: “*No information come to us*, *because if you knew that maybe he didn’t eat at noontime*, *maybe you can do something*;” “*In the project*, *there was a part the doctors filled in*, *and there was a part of the project where the parents filled out* [*…*] *what he did and didn’t do*, *according to us*, *and these observations had a weight*; *we had the feeling that our observations had a weight and that they always kept them in mind.*”

Mothers highlight a thematic area relating to interpersonal relationships within the family to which two issues belong. The first issue pertains to the relationship among siblings; the second issue concerns the relationship with the extended family, and in particular, with grandparents. The attention that mothers express about child’s siblings underlines the suffering that accompanies having a disabled brother and the conflict that characterizes the relationship: “*It also made me uneasy that A.* (*sister*) *had a disabled brother*; *now I would have a third child*, *because I think it right that my daughter also has a relationship with a brother*, *in quotation marks*, *able-bodied*, *a fair relationship*;” “*S.* (*sister*) *had a crisis*, *she started having fears* [*…*] *as a super-safe girl*, *she had become a girl full of fears* [*…*] *for her*, *her brother is important*, *even too much!*;” “*For L.* (*sister*), *a brother with a rare disease is the worst thing that ever happened to her*; *she always attacked him a lot.*”

Finally, problems with the family of origin are often narrated by women: “*We clash in the ways in which we think of others*, *not only relatives but also the narrow family*;” “*I would say that grandparents have begun to accept her now…to accept the disease*, *in the sense that they initially refused this thing*.”

### Social Support Perception

A second part of the interview was dedicated to the clarification of the type of social support mothers and fathers perceived, with particular attention to gender differences.

The content of the categories that emerge on the subject of perceived social support are described below. To simplify the reading of the data the representative quotes have been inserted in the Table 2. When the quotations are similar between fathers and mothers, we chose both quotations to better underline the conformity in meaning; when the issue is not present in interviews of mothers or fathers, there is a blank space.

**TABLE 2:** Social support perception in mothers’ and fathers’ narratives.

		**Fathers’ quotations**	**Mothers’ quotations**
Personal growth	Self-awareness		*It has opened a window on my inner world*
	Optimism	*It helped me to see positive things too*	
Emotional support	Understanding	*When you talk*, *you know you’re understood*	*We speak the same language that comes from the same kind of suffering*
	Sharing emotion	*It has always been useful to share feelings and ideas*	*It was useful to be able to express certain angers*, *certain misunderstandings*
	Leisure		*There were beautiful moments of joy*, *moments in which I felt the desire to have fun*, *to have moments of serenity between us*, *normal moments*, *the desire to feel happy*
Informational support	Suggestions	*The people in the group helped me because they taught me what to say*, *how to do it*	*We have practical indications on what we do*, *how we handle the subject of sterilization at home*, *for example*
	Experience	*In the group*, *there are people with much more experience than me who can help me*	*The experiences you have in the group become yours*, *like a small amount of experience*
	Comparison	*If we look at the children of colleagues*, *they are all graduates*, *engineers*, *all highly sought after by companies*, *excellent in everything*; *it seems to us that only we have this reality*, *this nightmare…instead*, *in the group*, *we see that we are not alone*	*It didn’t just happen to me*; *it also happened to others*, *and*, *in the end*, *L.* (*daughter*) *is better off than others*
Social function	Visibility	*I created a website to talk about the syndrome*	*One of the fundamental things was to have visibility*; *together*, *we could find transversal topics of interest*
	Social action	*I participated in writing* “*The White Book*,” *which denounces a situation of disadvantage to the institutions*, *the heavy situations that we live*	*We work to make institutions understand how rare these patients are and what needs they can have*, *starting with basic things like dental devices*, *which are not an esthetic cure but rather a fundamental support*

The analysis of the social support perceived by mothers and fathers highlights four fundamental thematic areas through which the members draw support from the self-help group (Table 2): personal growth, emotional support, informational support and the possibility of social action. Therefore, the self-help group represents the main source of social support for all respondents, and it becomes a fundamental reference point with which to share the pains as well as the successes related to the caregiving of a child with a rare disease, as this mother reports: “*When S.* (*daughter*) *started to walk alone*, *I was happy*, *and the first thing I thought was to celebrate with the group*” (mother).

For mothers, personal growth is characterized by the possibility of increasing self-knowledge and awareness, while for fathers, it appears to be more tied to the possibility of changing one’s point of view and beliefs about the situation.

The feeling of being understood and the sharing of emotions seem to characterize the emotional support received from both mothers and fathers. Women also perceive the possibility of experiencing moments of celebration in which they could be joyful together as a significant aspect of emotional support.

Informational support includes practical suggestions and experiences that are shared among participants in the self-help group. These aspects appear central to both mothers’ and fathers’ perceptions as a possibility of making a social comparison with others that can reduce the sense of loneliness.

Finally, the self-help group performs an important social function for both men and women, linked to the possibility of having visibility and organizing social actions. With respect to this specificity of social support, the theme of diagnosis appears to be central in the narratives of both the fathers and the mothers. The group seems to represent a key element in supporting the difficult search for a diagnosis: “*If I had not met X*, *my son would not be diagnosed*, *and if he had no diagnosis*, *it would be a serious problem for the family*” (father); “*The group urged me to find the diagnosis*” (mother).

## Discussion

Even if the literature has paid increasing attention to rare diseases, the specific aspect of gender differences in conditions such as caregiving for a child with a rare disease has not been examined as comprehensively. This work provides an original contribution to the field to clarify the complexity and the characteristics of caregiving for a child with a rare disease and to explore the specific gender differences regarding this topic. The present research is the first of its kind conducted in Italy and one of the few studies on caregiving that consider fathers’ points of view.

The findings suggest that a common area of crisis that characterizes mothers and fathers and that concerns diagnosis research and difficulties in communicating with healthcare professionals. Diagnostic delays, failure of diagnosis, and misdiagnosis represent a central theme in the studies on rare diseases and the European Organization of Rare Diseases has recently documented these difficulties in a report (Eurordis., 2009). According to van Van der Koolt et al. (2010) the lack of a prompt diagnosis may lead to a loss of confidence in the healthcare system. This may suggest that these two themes are so significant for caregivers to appear regardless of the syndrome and the gender.

However, there are some gender specificities, such as mothers’ greater interference and limitations in their work and social life due to caregiving. Furthermore, women are expected to adopt the role of caregivers, while men are not (Migliorini and De Piccoli, 2019). For mothers, the hardness of the disease, once known, emerges as a source of change with respect to their social lives. On the one hand, this change poses limits with respect to job opportunities, but on the other hand, it allows for the discovery of creative parts of the self and the opportunity to take on an instructional role in the positive rehabilitation of the child. However, the uncommon behaviors and lifestyles of patients with rare diseases are a continuous exercise for the family; in particular, the mother underlines the impact on siblings and grandparents, which implies a rethinking of the whole system. Contrary to a previous study (dos Santos et al., 2017), fathers do not express worries about siblings.

Uncertainty about the future, which is common among other parents of chronically ill children (Coffey, 2006; Emiliani et al., 2010), was also evident in these parents’ discourse, but in the case of a rare disease, this uncertainty is increased due to the lack of information about the illness and its therapy.

The focus on a diagnosis, which is a central theme of the present study, becomes even more important if it is connected to the theme of health innovation and to new possibilities introduced by genetic and scientific advances as well as new technologies to have more sensible and timely diagnoses. This change urges a deep understanding of the psychological effects of genetic diagnosis on individuals and their families (Rania and Migliorini, 2015; Alby et al., 2017; Battistuzzi et al., 2019).

In particular, worries about the future are a significant theme for fathers, while mothers are more focused on presents. This difference seems to reflect the diverse psychological functions that mothers and fathers exercise in growth. The different roles of education also appear to be consistent with those found in other studies in the Italian context, underscoring a new image of paternity as more involved in the care of children but mainly in recreational and executive activities (Alby et al., 2014; Rania et al., 2015a; Rebora and Rania, 2017).

The multitude of professionals and services involved constitute a fragmented framework that does not favor the necessary coordination between interventions, made even more difficult by the exceptionality of the symptoms and the ambiguity of the diagnosis that often characterize rare diseases. This framework heightens the perception of a lack of information and coordination in the health care system. This uncertainty is experienced with discomfort by the parents, also due to the absolute lack of indications, not only with regard to the characteristics of the disease itself but also with respect to the therapies, which are often non-existent. The literature in the Italian context emphasizes the importance to set up a multidisciplinary working group to diagnose and properly treat patients (Bizzi et al., 2016), our study suggests that this same attention should also be transferred to the whole family. Furthermore, family members seem to need to know the correct way to behave with their children. Often, doctors have no answers and can hide their difficulty behind technical communications, as evidenced by some testimonies. In line with previous works (Kohlschütter and van den Bussche, 2019) our study emphasizes the importance of supporting the role and competence of parents in the dialogue with doctors. From an operational point of view the results should encourage professionals to provide information in a language understandable to parents, supporting their involvement and their active participation. Indeed, the information received from other families is considered by parents to be more credible than that given by doctors. Communication with health professionals is a critical element in various areas of the doctor-patient relationship (Rania et al., 2015b, 2018; Rania, 2019); in particular, in the present study, this difficulty seems to considerably increase parents’ feelings of loneliness, which are already substantial because very few cases of the rare disease exist in Italy and abroad. In fact, it seems to be very important to know other families in the same situation to know how to face the problem.

The coordination task is left to the family, as it is the only component connected with all of the other components (Baumbusch et al., 2018; Currie and Szabo, 2018). Parents of a child with a rare disease must coordinate a series of fundamental activities for the management of the disease and must engage to obtain the correct diagnosis and the consequent rights necessary to access the various social support services and navigate bureaucratic difficulties. Parents often have as much, if not more, knowledge of their child’s diagnosis than do healthcare providers, so they consider themselves experts (Brewer et al., 2008).

The present work underlines that caring a child with a rare disease is extremely challenging because people with rare diseases are often invisible (Čagalj et al., 2018); consequently, their parents are also invisible. Parents do not perceive themselves as potential users of the formal support system and look for an answer to their needs through their relationships with peers.

Our study shows how the self-help group could serve as the key operator when it provides parents with feedback and information, helps in the interpretation of medical communications, and facilitates the identification of new perspectives and the evolution of problems.

The self-help group helps participants feel less isolated, both in terms of closeness (the part dedicated to sharing experiences) and in terms of practical support. In fact, the social activity of the group can offer visibility to parents and their situations; they would not have been able to have such visibility otherwise.

Knowing how to grasp the emotional resonance that others’ stories achieve within ourselves and then being able to share them allows us to achieve mutual help. The group, through the various forms of feedback offered and the possibility of comparison, favors the acquisition of skills and attitudes more effectively toward the shared situation. The self-help group in this study is a significant reference for emotional support and practical assistance, serves a social function (Dennis, 2003), and aids in personal growth. These aspects allowed the members to perceive a communality in their experiences, despite significant diversity of syndromes. According to previous study (Finlay et al., 2018) support group could “emphasizes belonging and interpersonal engagement to initiate stability within the group, minimizing difference and maximizing communal coping” (p. 864).

This offers significant insight that could encourage clinicians to promote participation to self help group for parents of children with rare disease. The possibility that the group seems to offer to fathers to look at their lives with optimism is inserted among the qualities that specifically characterize resilience (Walsh, 2006). The prospective of resilience applied to families shifts the focus from a family seen as damaged to a family seen as “challenged” and is based on the belief that individual and family development can be forged by collaborative efforts to face adversity.

According to previous work (De Piccoli et al., 2016), the results underline that it is necessary to reduce the gender stereotypes that are still present in parents’ perceptions to promote a greater balance in caregiving. Therefore, the importance of gender perspectives in health science is increasing in last years (Migliorini and De Piccoli, 2019; Migliorini et al., 2019).

Finally, a greater understanding of the difficulties these families face could lead to improved service delivery to these families, considering both the general needs of the family and the individual needs of each family member according to gender.

## Limitations and Further Implication

There were, of course, some limitations to this study. First, the temporal variable was not considered with respect to the communication of the diagnosis. The literature underlines that over time, changes can occur in individuals’ emotional feelings and internal planning with regard to the diagnosis (Houlis et al., 2018). In the future, it might be interesting to study the sources of support that activate parents who do not participate in a self-help group to see how needs and prospects change. Another limitation could be the variety of different rare diseases that parents have to face, however, the condition of rarity of the disease is common to all parents and literature underline that is this condition that lead to difficulties that victims of more common disorders don’t encounter (Van der Koolt et al., 2010).

Furthermore, it could be useful to analyze the role of siblings in intrafamily support, with special attention to how family routines change after diagnosis (Hammons and Fiese, 2010). Regarding research implication it will be worthwhile to further investigate the different perception of mothers and fathers about social support and family daily workload using measures like the Ecocultural Family Interview that has already been used in Italian context (Axia, 2004).

However, this study attempts to fill a gap in the literature because the experiences of men providing care have not been adequately explored (Sharma et al., 2016).

## Data Availability

The datasets generated for this study are available on request to the corresponding author.

## Ethics Statement

This research was conducted following the ethical norms stipulated by the AIP (Italian Psychology Association). Before the interview, written informed consent was obtained from all the participants in accordance with the Declaration of Helsinki. It contained a brief explanation about the research and informed potential participants that the interview would be audio-recorded and the data processed and anonymized; it also assigned a code to each participant, in compliance with Italian Law on Privacy no. 196/2003. Research ethic committee has not yet established in the authors’ institution when the research started, so an ethics approval was not required for this research as per the authors’ Institutions’ guidelines and national regulations.

## Author Contributions

NR conceived of the presented idea and supervised the findings of this work. PC developed the theory, performed the qualitative analyses, and wrote the manuscript with support from LM. All authors discussed the results and contributed to the final manuscript.

## Conflict of Interest Statement

The authors declare that the research was conducted in the absence of any commercial or financial relationships that could be construed as a potential conflict of interest.
